# Small Animal Shanoir (SAS) A Cloud-Based Solution for Managing Preclinical MR Brain Imaging Studies

**DOI:** 10.3389/fninf.2020.00020

**Published:** 2020-05-19

**Authors:** Michael Kain, Marjolaine Bodin, Simon Loury, Yao Chi, Julien Louis, Mathieu Simon, Julien Lamy, Christian Barillot, Michel Dojat

**Affiliations:** ^1^INRIA U1228, INSERM, Université de Rennes, Rennes, France; ^2^INSERM U1216, Grenoble Institut des Neurosciences, Université Grenoble Alpes, CHU Grenoble Alpes, Grenoble, France; ^3^ICube, University of Strasbourg-CNRS, Strasbourg, France

**Keywords:** MRI, data sharing, open science, population imaging, image processing

## Abstract

Clinical multicenter imaging studies are frequent and rely on a wide range of existing tools for sharing data and processing pipelines. This is not the case for preclinical (small animal) studies. Animal population imaging is still in infancy, especially because a complete standardization and control of initial conditions in animal models across labs is still difficult and few studies aim at standardization of acquisition and post-processing techniques. Clearly, there is a need of appropriate tools for the management and sharing of data, post-processing and analysis methods dedicated to small animal imaging. Solutions developed for Human imaging studies cannot be directly applied to this specific domain. In this paper, we present the Small Animal Shanoir (SAS) solution for supporting animal population imaging using tools compatible with open data. The integration of automated workflow tools ensures accessibility and reproducibility of research outputs. By sharing data and imaging processing tools, hosted by SAS, we promote data preparation and tools for reproducibility and reuse, and participation in multicenter or replication “*open science*” studies contributing to the improvement of quality science in preclinical domain. SAS is a first step for promoting open science for small animal imaging and a contribution to the valorization of data and pipelines of reference.

## Introduction

Recently, the validity of the published results in life sciences has been questioned (Button et al., 2013; Ingre, 2013). The poor replication of research findings is directly linked to an endemic low statistical power of the majority of biological studies. It is now well recognized that data sharing should be promoted to improve the robustness and the replicability of the scientific results (Poldrack et al., 2017). Technical solutions should be proposed to support a more “*open science*” approach, making data discoverable, accessible, interpretable, and reusable, as captured in the FAIR Guiding Principles (Findability, Accessibility, Interoperability, and Reusability) (Wilkinson et al., 2016). For human population imaging, large data repositories exist, such as ADNI for Alzheimer disease, PPMI for Parkinson disease, centerTBI for brain trauma, United Kingdom Biobank for various diseases or ConnectomeDB for the Human Connectome Project, extensively used by researchers all over the world and leading to new findings. Additionally, data analysis plays an important role. For such large datasets, image processing pipelines should be constructed based on the best algorithms available and their performance should be objectively compared to diffuse the more relevant solutions. Also, provenance of processed data should be secured (Mackenzie-Graham et al., 2008). Specific architectures (e.g., COINS, LONI) are now available mainly for human population imaging [see (Kennedy et al., 2019) and other recent works here].

Similarly, to human population imaging, there are several well-founded motivations for *animal population imaging*: optimization of costs and subject participation, improvement of quality of science (use of sufficiently large animal cohorts for ensuring statistical result validity, e.g., drug development process) and enhancement of research discovery [see the special Lab Animal focus on reproducibility in animal research (Prescott and Lidster, 2017)]. However, at the difference of Human imaging, where the issues with large cohorts are well-known and solutions have been proposed, sharing small animal imaging data and corresponding image processing pipelines is a recent preoccupation. Some efforts are performed especially toward the construction of animal atlases (Sunkin et al., 2013; Bjerke et al., 2018; Wang et al., 2018). Despite of some attempts (Lee et al., 2010), there is a clear miss of appropriate infrastructure for facilitating sharing data and processing tools for animal population imaging. Indeed, solutions developed for Human studies can only be reused in part and should be adapted and extended to take into account the specificities of animal studies: for instance, with the introduction, in the data model, of new concepts for strain and anesthetic, or of a specific converter for raw data (e.g., mainly Bruker files). Moreover, population imaging infrastructures for clinical or preclinical purposes, have to handle, presently or in a close future, very large datasets while providing a fast and robust data access via an efficient web interface. Today the proposed solutions do not really address this issue because internal mechanisms for scalability are missing. Presently, as an example, we currently handle clinical studies with up to 3000 subjects with around 4Tb of data and we are now just at the emergence of the population imaging studies, recently boosted by the introduction in life science domain of efficient machine learning approaches, avid of large mass of data.

In this paper, we present the Small Animal Shanoir (SAS) solution. SAS is an extension of the Shanoir data management system for Human brain imaging repositories (Barillot et al., 2016) with the integration of dedicated microservices for small animal imaging studies. SAS was developed under the hospices of the national infrastructure project France Life Imaging, with the goal of coordinating research activities in France in *in vivo* imaging and combining the skills to push the current technological barriers. The specific action “Information Analysis and Management” (FLI-IAM) was devoted to the development of a federated infrastructure for the management of *in vivo* imaging data and processing tools. FLI-IAM is partner of OpenAIRE-connect, an H2020 European project promoting Open Science.^1^ The paper is organized as follows: firstly, the global architecture of FLI-IAM is presented, then SAS and the notion of micro-service are introduced, illustrated by the description of a selection of representative micro-services; then current applications are briefly presented followed by the discussion section.

## Architecture Overview

**FIGURE 1:**
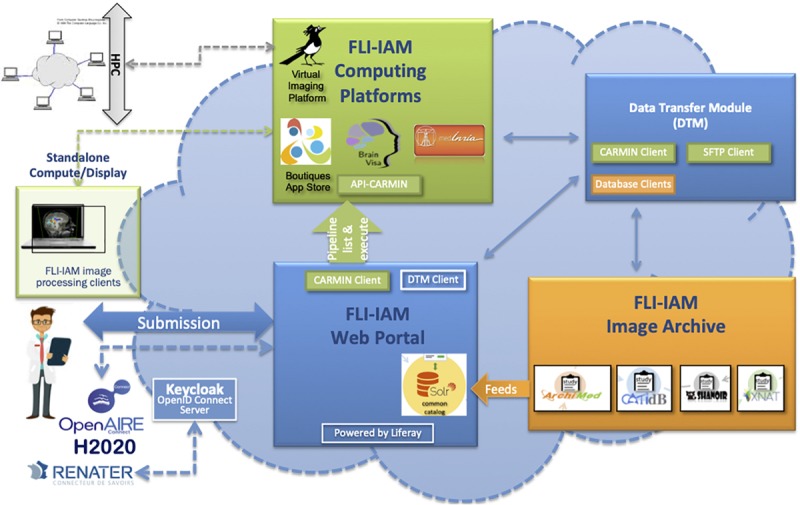
The FLI-IAM architecture. Three major blocks compose the core of this distributed web architecture: the web portal (in blue), the image archive (in orange) and the computing platforms (in green). The data transfer module (in blue) ensures data exchange between these three modules. The API-CARMIN allows the seamless connection of data and pipelines. The user submits a query via the web portal (e.g., selecting images following criteria and choosing the execution of an imaging processing pipeline). The user’s identification can be done via a Renater account or realized via OpenID connect implemented using Keycloak. Presently, four databases and four computing platforms are federated by the infrastructure. The integration of a new database is realized by the introduction of a connector that maps its data model to the common catalog. Each database manages autonomously multicenter imaging studies. Several instances of each database can be managed be the infrastructure. Pipelines can be executed on high performance computing cluster (HPC) or on a local machine. This DTM module ensures the transfer of data between a database (dedicated clients in orange) and the corresponding computing resource via the API-CARMIN. A local file system and database are used for a temporarily storage. Each platform (database or computing resource) can be configured with its web address and each transfer can be started and monitored. For each transfer between two platforms, a communication channel (protocol) can be chosen. A scheduler assures long-time data transfers. The architecture is part of the H2020 European OpenAire project for open science promotion.

The goal of the architecture is to federate different systems that have their specific data model which does not necessary fulfill the XNAT reference scheme. For this purpose, we chose to use the core ontology OntoNeurolog, accessible via BioPortal.^2^ This ontology is based at the upper level on the DOLCE foundational ontology and described in details in Temal et al. (2008); Gibaud et al. (2011); Batrancourt et al. (2015). All specific data models including XNAT are mapped to this core ontology. The technical architecture of FLI-IAM is shown in Figure 1. The FLI-IAM architecture is web-oriented and constructed on three main functionalities: (1) query and retrieval of data and image processing pipelines (via the web portal), (2) storage of data and associated metadata (via the image archive module), and (3) the execution of the selected pipelines on the selected data (via the computing platforms module).

The data transfer module (DTM) is used to exchange data between the image archive and the computing platforms. The architecture was successfully used for hosting two MICCAI Human imaging challenges on Multiple Sclerosis (Commowick et al., 2018) and PET tumor segmentation (Hatt et al., 2018).

### FLI-IAM Web Portal

The web portal of FLI-IAM^3^ guides the user to the resource or service adapted to his/her needs. Therefore, an overview of all resources and services available is proposed. After registration and authentication, the candidate accesses to the set of components: the image archive via the common catalog graphical interface, the computing platforms via the CARMIN client (see below) and the DTM via the DTM client. Technically the FLI-IAM web portal is based on Liferay Portal Server v6.2, Community Edition, a Java Enterprise application server that provides a full content management system and a market place with multiple extensions. For the MICCAI challenges the portal was used to inform about the challenge rules, register as candidate, with a process specifically integrated for this purpose, and give access to the data.

#### FLI-IAM Authentication Process

Security is a sensitive aspect for all web-oriented data management systems. OpenID Connect (OIDC) is the authentication protocol used by the FLI-IAM infrastructure, implemented in Keycloak, to verify the identity of a user based on the authentication performed by an authorization server, as well as to obtain basic profile information about the user in an interoperable and REST-like manner (see Figure 1). All components in FLI-IAM delegate the authentication to Keycloak that allows single sign-on with identity and access management to applications and services. In line with GDPR, all user passwords are exclusively stored in Keycloak and only the current user has knowledge of his/her password. For academic researchers who have a Renater account, a connection between FLI-IAM Keycloak with Renater is always possible with the SAML authentication provider.

#### Common Catalog

**FIGURE 2:**
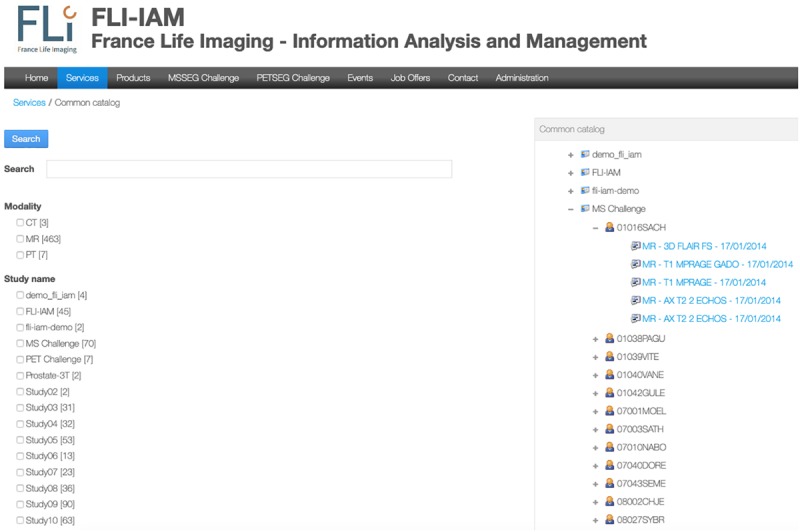
User’s interface for either a specific search query or to display all images available for one user. In this example, no specific query is addressed and the interface shows all the available studies. An expanded view on T1-weighted images for the MICCAI MS challenge is displayed.

FLI-IAM portal users can execute cross-database searches to find published datasets and studies in the different databases using the common catalog. A free search-field and a search result display page are provided within the graphical user interface of the portal (see Figure 2) thanks to the search engine Apache Solr.

A specific workflow for the combination of metadata has been developed for the whole interrogation of the set of databases. Imaging studies, associated meta-data and corresponding images are hosted within each instance of the federated databases. Each database manages independently its users, access rights and roles for the hosted studies. The Principal Investigator (PI) of a study has the responsibility to place metadata into the common catalog of the FLI-IAM infrastructure for sharing the study publicly or with specific user groups, using access control lists. Users of the portal can then identify the origin of the datasets and ask the corresponding PI for data access. Technically a common search index has been defined and four mapping plugins have been developed, used for the connection to the common catalog of the corresponding database instance. Once a PI has allowed data sharing of a study, a nightly job updates the common catalog. When he/she decides the sharing ending, the corresponding datasets are deleted from the search index.

### FLI-IAM Image Archive

The FLI-IAM infrastructure federates currently four imaging databases. Their main features are described in Table 1. CATI-DB and Shanoir manage various large mono- or multicenter brain studies, while ArchiMed (Micard et al., 2016) collects imaging data from various clinical research projects. Presently, each database is hosted on an independent hardware infrastructure and could be hosted in a future central FLI-IAM hardware infrastructure. Each database has its independent workflow to import images. The federative architecture is constructed for a seamless integration of new databases.

**TABLE 1:** Main characteristics of the four databases currently hosted by FLI-IAM.

**Database**	**Who**	**Key concepts**	**Modalities**	**Browser**	**Add. Info**	**Open source**
ArchiMed	CIC-IT, Nancy, Inserm, Fr	Centralized server, Model close to DICOM/PACS, Desktop client, PAAS	MRI, CT	No	Link	Not yet
CATI-DB	CATI, Paris, CEA, Fr	Centralized file-system (NFS) with meta-data server, Model oriented on processing, Perfectly adapted into CATI-infrastructure, SAAS (turn-key)	MRI, PET/SPECT	No	Link	Yes, access registration
Shanoir	Empenn, Rennes, Inria, Fr	Centralized server, Ontology based, Model remains fix, Web client, PAAS	MRI, PET, CT, EEG	Yes	Link	Yes, on GitHub
XNAT	NRG Lab, Washington, University School of Medicine, US	Centralized server, Schema based, Default model to customize, Web client, PAAS	MRI, PET, CT	Yes	Link	Yes, on Bitbucket

*PAAS, Platform As A Service; SAAS, Software As A Service. MRI, Magnetic Resonance Imaging; CT, Computed Tomography, PET, Position Emission Tomography: SPECT, Single-Photon Emission Computed Tomography; EEG, Electro-Encephalography.*

For a common interrogation of the federated databases, an excerpt of the common catalog is presented in Table 2.

**TABLE 2:** Common catalog. A common catalog is required for querying the different databases accessible via the federated platform.

**Common catalog**	**ArchiMed**	**CATI-DB**	**Shanoir**	**XNAT**
study_name	study_code	Study.name	Study_Name	projectData.name
center_name	institution_name	Center.name	Center_Id	experimentData.acquisitionSite
manufacturer	manufacturer + manufacturer_model	unknown	Manufacturer_Name + Manufacturer_Model	imageSessionData.scanner
principal_investigator	study_investigator	Study.main_investigator	Investigator_Id	experimentData.investigator
subject_identifier	unknown in db, examCode used	Subject.code_in_study	Subject_Name	subjectData.ID
series_modality	file_type_acronym	Fileset.attribute.modality	Dataset_Ref_Dataset _Modality_Type_Id	imageScanData.modality
series_protocol	serie_desc	Fileset.attribute.sequence	Dataset_Name	imageScanData.series_description
series_creation_date	serie_date_time	Assessment.date	Dataset_Creation_Date	imageSessionData.date

*The table above describes the structure of the common index and the mapping of each database into the common catalog.*

### Computing Platforms

As shown in Figure 1, beside databases, FLI-IAM integrates four computing platforms: BrainVisa, medInria, Virtual Imaging Platform (Glatard et al., 2013) and Boutiques (Glatard et al., 2018). The former, BrainVisa is a neuroimaging software platform for mass data analysis, providing image processing tools such as Morphologist for brain segmentation and sulcal morphometry, and an interactive 3D neuroimaging data visualizer (Anatomist). medInria is a multi-platform medical image processing and visualization software. Through a graphical user interface, medInria offers processing tools for medical images, such as 2D/3D/4D image visualization, image registration, diffusion MR processing and fiber tracking. VIP is a web portal for the execution of medical image processing pipelines for massive data analysis and simulation. It leverages resources available in the biomed virtual organization of the European Grid Infrastructure to offer an open service to academic researchers worldwide. Boutiques represents the app store of FLI-IAM. Boutiques allows automatic publication, integration and execution of applications across computational platforms. Boutiques applications are described in a JSON file and enable the simulation, validation, evaluation, and application-specific monitoring of command-line tools. Providers can integrate their application into a Docker or Singularity container, describe it and share it via Boutiques. Each computing platform can be used and installed independently. Note that Boutiques is integrated into VIP.

FLI-IAM provides an integration layer with two features: (1) an authentication using OpenID Connect and (2) a unified remote access via the REST-API, CARMIN. Using such an environment, the same client can remotely use each platform implementing the interface. For example, this allows the FLI-IAM web portal, via the CARMIN client (JavaScript library), to access to a list of pipelines provided by VIP, send data and execute a pipeline. Moreover, the same CARMIN client can be integrated into each of the database instances present in the FLI-IAM image archive for its connection to any computing platform. Note that CARMIN is also implemented in CBrain, a Canadian infrastructure used for managing very large imaging medical projects. Thus, the users of the FLI-IAM web portal have potentially access to CBrain and *vice versa*.

### Data Transfer Module

The DTM connects the image archive with the computing platforms. It transfers primarily images from one database (source) to one computing platform (destination), but can as well transfer images between databases or between computing platforms. Additionally, DTM can call a computing platform, e.g., VIP, to start processing pipelines with a given pipeline identifier, check for computation results and import the processed images back into one of the databases (see Figure 1). Therefore, it integrates four client libraries (one for each database), a SFTP client and a CARMIN client. The DTM is completely configurable on using its web interface and has been used for the MICCAI 2016 challenges. A MySQL database is used to maintain state and all transferred data are temporarily stored in a local file system.

## Small Animal Shanoir

Small Animal Shanoir is built on the top of a recent version of Shanoir, called Shanoir-NG, which relies on recent web technologies, such as Single-Page-Applications (SPAs) and micro-services, with the double objective of reinforcing the flexibility and scalability of the architecture.

### Single-Page Application

Shanoir-NG has been developed as SPA for two major reasons: first to give the user the feeling of interacting with a desktop application rather than with a web application; second to support as many browsers as possible, even mobile browsers. This is based on the assumption, that modern modular SPAs could be more easily adapted to reduced screen sizes and other requirements of mobile applications. Technically SPAs rewrite parts of a page rather than loading the entire page and the application code is completely loaded when the application starts. Appropriate resources are dynamically loaded and added to the page as necessary, usually in response to actions of the user. Angular five has been chosen as front-end technology after an evaluation of different frameworks available yet.

### Micro-Service Architecture

Inspired by the multi-agent architecture (Minsky, 1986), the micro-service architecture^4^ considers the application as a suite of independent services; each service running with its own process and communicating with lightweight mechanisms, often an HTTP resource API. The main advantage of adopting a micro-architecture is the scalability in case of evolving computational load or availability of hardware resources. Moreover, the strong independency of services allows for their development and deployment separately from the others ensuring a continuous service delivery. Each micro-service can be developed using the more efficient language for its functionality with its own memory management, as long as the common external interface policy is respected.

The strong independency of services requires the improvement of the control at the global level of the architecture. Some control concerns are particularly important for our applications: for instance, how does react a micro-service when another depending service is no more available? Or how each micro-service is informed about changes in its environment or security policy? Some inconsistencies have to be tolerated for a short period of time and handled afterward, when a micro-service, stopped for a while, is back to function. Specific errors or activity tracing mechanisms have to be introduced to trace the service states, user actions and other events.

As a Cloud-native application, each micro-service is integrated in a Docker container. For instance, the DTM micro-service described above runs its own Docker container, provides a REST-API and an Angular five front-end to configure available platforms, connection settings (channels) or initiate/monitor transfers. The entire Shanoir-NG architecture is deployed using a Docker Compose script that initiates and runs each Docker container encapsulating a micro-service.

Based on the application specifications, we have to define how to distribute the different functionalities into a reasonable collection of micro-services, while preserving a good integration. Figure 1 in Supplementary Material shows the current architecture. Six key services have been identified so far Users, Studies, Import, Datasets, Preclinical and Dicomifier. The two-latter specific for small animal applications are detailed below.

### Preclinical Micro-Services

**FIGURE 3:**
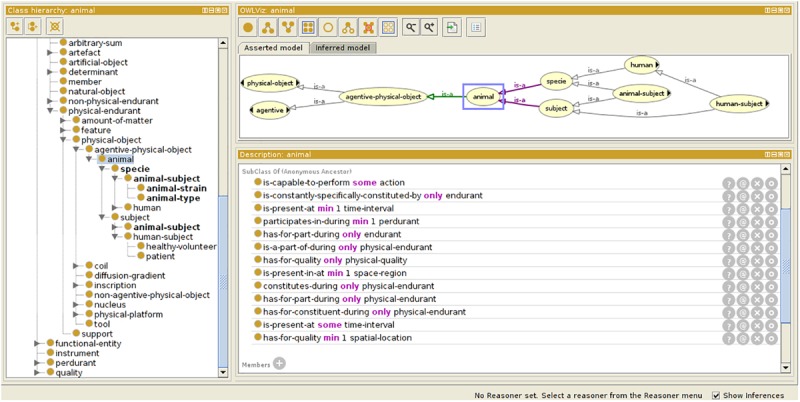
Ontology extension for preclinical study. Specific concepts are introduced extending the OntoNeurolog ontology expressed in OWL and represented using Protégé (https://protege.stanford.edu/about.php).

For preclinical applications, the initial OntoNeurolog ontology was extended by the preclinical working group of FLI-IAM (SAIN) to introduce several concepts related to animal study for facilitating their sharing (see Figure 3). Thus, concepts have been added to describe the subject (specie, strain, provider, type [transgenic or not], and stabling), the model of pathology under study and the administered therapy. The experimental conditions are also described (type of anesthetic, physiological measurements: e.g., blood gas, temperature, SaO2, heart rate, …). The ontology is represented in OWL using Protégé (Musen, 2015).

**FIGURE 4:**
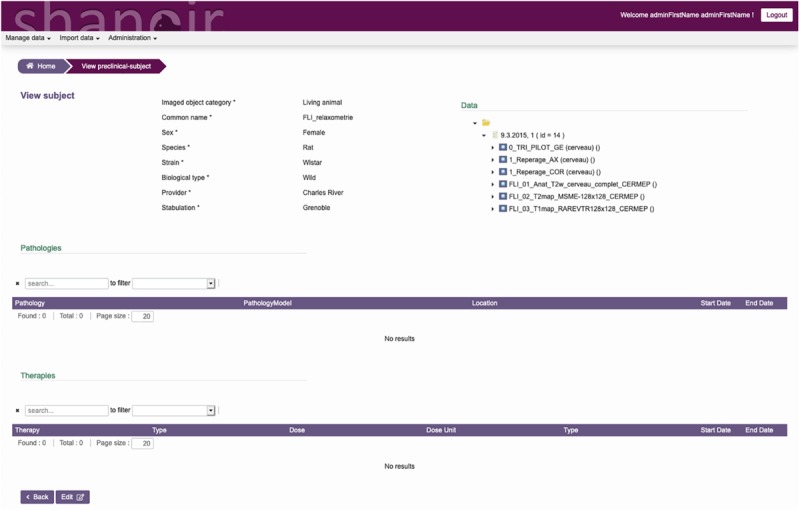
SAS, GUI for animal studies, information about the subject. Pathologies and therapies can be added and managed.

**FIGURE 5:**
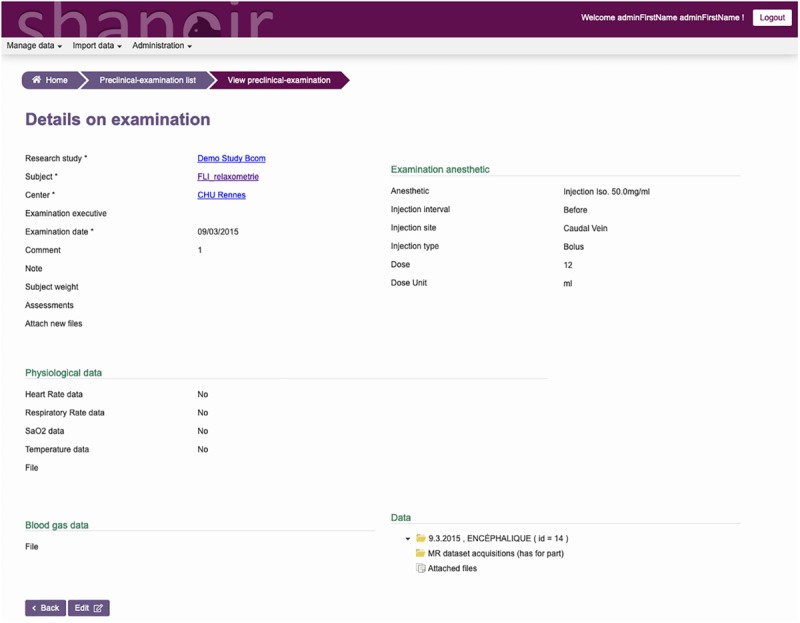
SAS, GUI for animal studies, details about the experimental conditions. Anesthetic, physiological, and blood gas data can be added and managed.

The ontological preclinical model has been translated into a relational database model and integrated into the preclinical microservice. It manages its own graphical user interface (GUI) and database and was added to Shanoir-NG. Figures 4, 5 show the corresponding dedicated interface.

### Dicomifier Micro-Service

Dicomifier is a generic and open-source converter designed to process both preclinical and clinical imaging data and to be easily extensible to account for the wealth of meta-data associated with medical images. This micro-service converts the current imaging data formats, Bruker and DICOM files. The former is largely used in preclinical imaging centers because the same manufacturer (Bruker Biospin) designs the preclinical MR scanners. The converter is modular: one part handles the conversion from the Bruker format to the DICOM format and another part handles the conversion from the DICOM format (either native or converted from the Bruker format) to NIfTI. This design allows future extensions, e.g., conversion from NIfTI to DICOM to store segmentation results in a PACS. Similar concepts guiding the hierarchy of data exist in both Bruker and DICOM formats: patient (or subject), study (i.e., a set of exams performed for a single goal) and series (single or multiple 2D or 3D images from one acquisition). However, implementation of those concepts in both formats differs, and, in addition to the file-format difference, data alignment is an integral part of the conversion. The Bruker-DICOM alignment is a mapping process in which a pre-defined, modality-specific, set of DICOM elements is extracted from the Bruker-format exam. The mapping may be a direct one, i.e., mapping a single Bruker element to a single DICOM element without additional transforms, or a more complex one, using data from multiple Bruker fields, transforming them, and storing them in disjoint DICOM fields.

Regarding the NIfTI conversion, we leverage the multi-dimensional capabilities of NIfTI to store images per-series, following the dimensions given by the DICOM files. For instance, a diffusion MRI acquisition with multiple b-values would appear as a five-dimensional dataset. The fixed-size header of the NIfTI format cannot accommodate the wide range of meta-data stored in the DICOM format, although this data may nevertheless be required for further processing, e.g., in parametric MRI. We chose to store the metadata in the widespread JSON format, for its seamless handling by many software libraries while remaining human-readable. For native DICOM files, i.e., not converted from Bruker, a normalization step is also applied to parse the data from vendor-specific fields, when the syntax of those fields is known.

### Current SAS Applications

*In vivo* T1 and T2 relaxation times extracted from MR imaging data are tissue dependent and their modifications may be characteristic of specific anatomical regions and pathological situations. Recently, we used the SAS infrastructure to produce quantitative 3D T1 and T2 maps for the healthy rat brain at 7T. Such maps were defined based on data acquired at two different centers on twenty Sprague-Dawley rats at GIN (Grenoble, Fr) and twenty Sprague-Dawley rats at CRMBM (Marseille, Fr). We used three different fitting algorithms from three difference centers (CRMBM, MRICen (Fontenay-aux-roses, Fr) and Icube (Strasbourg, Fr) to compute the T1 and T2 values in each voxel and to evaluate the influence of image processing steps on the final maps. To derive relaxation time values per brain area, two multi-atlas segmentation pipelines from MIRCen and Icube were executed. All data were stored on SAS and image processing pipelines were embedded into Docker containers and executed on the VIP processing platform. With the DTM, processed data were stored in SAS for each individual.

**FIGURE 6:**
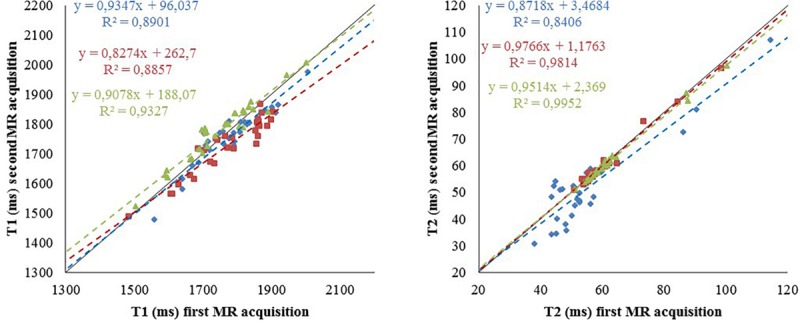
Reproducibility of the relaxometry maps. T1 **(left)** and T2 **(right)** values for the 29 regions of interest from data acquired at CRMBM (in blue) for one rat (S32) and at GIN for two rats (S21 and S22) in red and green, respectively. The pipelines were provided by CRMBM (fitting) and Icube (segmentation). Dashed line: linear regression, corresponding equation are indicated with the same color code as values, R^2^: correlation coefficient. Bold line identity curve.

This work investigated the influence of data acquisition centers (*n* = 2) and image processing pipelines (*n* = 3) on the production of T1 and T2 brain maps. Figure 6 shows the reproducibility of the T1 and T2 relaxometry values computed from two MR acquisitions for each rat in each center (intra-reproducibility) and the coherency between the values obtained between the centers (inter-reproducibility).

It showed the coherence of the final computed maps when the acquisition sequence parameters are optimized and harmonized between centers (Deruelle et al., 2020). Using SAS, the study demonstrates the feasibility of pooling animal data from different centers. The use of a distributed computing architecture for image storage and for processing pipeline execution paves the way for future multi-center preclinical studies. The quantitative reference maps produced for longitudinal (T1) and transverse (T2) relaxation times obtained on 40 rats (to our knowledge the largest number to date) are valuable to investigate and monitor deviations from normality in animal model of pathologies. A similar study at different magnetic field on mice is undergoing with the same infrastructure involving more data providers (12 centers).

## Discussion

Similarly to human research, sharing (data and tools) requirements will necessarily grow up in the next future for preclinical (animal) studies. The design of SAS was motivated by the needs of the preclinical research community. The problem of replication and reproducibility in preclinical studies is now well documented (Sena et al., 2010; Begley and Ellis, 2012; Tsilidis et al., 2013) and can negatively impacted the translation to human studies, leading to waste of resources, unnecessary risks for volunteers and slowing down new therapies development. The first cause of non-reproducibility is the weakness of experimental design including low power (Begley and Ioannidis, 2015).

With SAS, we show that animal population imaging is feasible and that research in this field can be conducted using tools compatible with open sharing and use of automated workflow tools to ensure accessibility and reproducibility of research outputs. Our first preliminary experiments (see §3.5) demonstrate the strong interest of the community to promote the federation of multiple sources of information, processing tools and diffusion of knowledge distributed in various centers for preclinical studies. We will promote data preparation and tools for reproducibility and reuse and participation in multicenter or replication studies. Thanks to the FLI-IAM architecture used, we will diffuse our results beyond the national community. Indeed, a recent external survey (30 interviews) indicated that the primary application domain for such an infrastructure was preclinical imaging where solution is missing. Note that developing such a platform requires some efforts: from 2016, up to 15 computer engineers were employed for Shanoir-NG and SAS developments.

Moreover, FLI-IAM is partner of OpenAIRE-connect, for connection with European large scale open science projects. Additionally, SAS will contribute to the promotion of this platform and in return will be internationally visible: data and tools will be interoperable and queryable via international platforms. In using SAS, users strongly advocate for the diffusion of acquired and processed data and dissemination of imaging processing pipelines. They promote *Open Science by Design* (Committee on Toward an Open Science Enterprise et al., 2017), meaning that research conducted openly and transparently leads to better science. SAS is a first step for promoting Open science for small animal imaging and a contribution to the valorization of data and pipelines of reference. SAS will also be a good starting point for launching specific working group for animal MR studies at the European (RDA) or International (INCF, ISMRM) levels creating a culture that actively supports Open science by Design.

### Extensions and Future Plans

As a cloud platform, multiple extensions are already defined in the roadmap of Shanoir-NG. From the operational point of view the following extensions will have to be addressed: continuous delivery, automatic service discovery, data replication, backup and constant monitoring. The usage of smaller, in terms of memory usage, Docker container base images should be evaluated to optimize the memory footprint of the entire application. With the extension of the number of micro-services, the use of new container management platforms such as Apache Mesos or Kubernetes should be envisaged. From the functional point of view, we plan extensions for massive data import and download and management of BIDS format.

Finally, our next step is to connect SAS, a tool dedicated for small animal imaging, to other complementary environments dedicated to genetics or biology. The idea is to seamlessly integrate SAS with the open science community and to provide researchers a tool for easy sharing in a controlled way.

Shanoir-NG and its extension SAS are open-source software, published under the GNU General Public License (GPL), version 3. The source code is freely available on GitHub under^5^. The current implementation of SAS is available at^6^. Developer documentation is centralized in the GitHub Wiki^7^ with code^8^ and general user information can be found under http://shanoir.org. The development of Shanoir-NG is driven by a roadmap that is defined by the members of the consortium Shanoir within InriaSoft. Contributions are integrated in form of pull requests. The source code of Dicomifier is available at^9^.

OpenID Connect (OIDC) is an authentication layer on the top of the authorization framework OAuth 2.0, a standardized protocol controlled by the OpenID Foundation. This protocol is implemented in Keycloak, an open source software product that implements the OpenID Connect protocol and then is used as the central authorization server.

Renater is a highly fast and secured academic European network for internet access and associated services.

SAML (Security Assertion Markup Language) defines a protocol for security information exchange. It is based on XML and proposes a unique (single sign-on) identifier.

Apache Solr is a search and navigation platform built on the open-source HTTP server, Apache.

Docker allows to define containers in a standardized way that encapsulate code and the associated dependencies for an execution on different computing environments.

Singularity is an alternative solution to Docker.

CARMIN (Common API for Research Medical Imaging Network) defines a REST-API for exchanging data and remotely calling pipelines provided by computing platforms. The REST-API is specified using the OpenAPI initiative (OAI) available on SwaggerHub.

## Data Availability Statement

The datasets generated for this study are available on request to the corresponding author.

## Author Contributions

MK, CB, and MD contributed conception and design of the study. MK, MB, SL, YC, JL, and MS implement the different micro services; MK, JL, and MD wrote the first draft of the manuscript. All authors contributed to manuscript revision, read and approved the submitted version.

## Conflict of Interest

The authors declare that the research was conducted in the absence of any commercial or financial relationships that could be construed as a potential conflict of interest.

## Footnotes

1 OpenAire aims at developing a technical infrastructure for an interoperable European network of research repositories through the establishment of common guidelines and shared metadata standards.

2 https://bioportal.bioontology.org/ontologies/ONL-DP

3 https://portal.fli-iam.irisa.fr/home

4 https://martinfowler.com/microservices/

5 https://github.com/fli-iam/shanoir-ng

6 https://shanoir-ng-dev.irisa.fr

7 https://github.com/fli-iam/shanoir-ng/wiki

8 https://github.com/fli-iam/shanoir-ng/tree/appning

9 https://github.com/lamyj/dicomifier
