# Supplementary material for: Small Animal Shanoir (SAS) A Cloud-Based Solution for Managing Preclinical MR Brain Imaging Studies
Source: Front Neuroinform. 2020 May 19;14:20. doi: 10.3389/fninf.2020.00020 (PMC7248267; doi:10.3389/fninf.2020.00020)
Supplement: Supplementary file 1 [file Presentation_1.pdf]

Figure 1: Microservice architecture of SAS. The introduction of microservices allows a more independent development of the entire application, as each microservice is responsible only for its functionalities. New functionality e.g. in form of a new microservice (see Dicomifier) can be added more easily. Four microservices in the middle (Users, Studies, Import and Datasets) build the core of the architecture. Preclinical and Dicomifier are two independent microservices which, added to the internal PACS dcm4chee-arc-light, form SAS, the extension of Shanoir-NG for small animal imaging applications.
