# Supplementary figures and images for: Small Animal Shanoir (SAS) A Cloud-Based Solution for Managing Preclinical MR Brain Imaging Studies
Source: Front Neuroinform. 2020 May 19;14:20. doi: 10.3389/fninf.2020.00020 (PMC7248267; doi:10.3389/fninf.2020.00020)

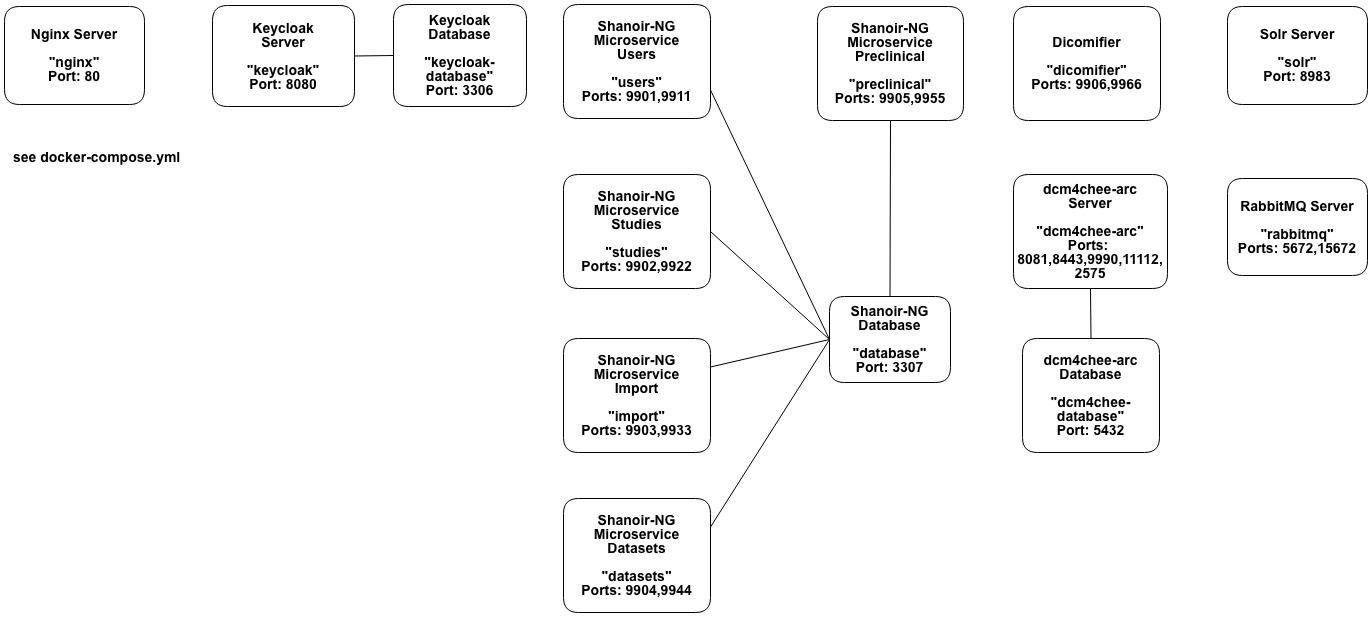

Supplement: Supplementary file 2 [file Image_1.jpeg]
